# Repetitive transcranial magnetic stimulation reduces smoking cravings by decreasing cerebral blood flow in the dorsolateral prefrontal cortex

**DOI:** 10.1093/braincomms/fcaf101

**Published:** 2025-03-05

**Authors:** Shuang Li, Anhang Jiang, Xuefeng Ma, Bo Yang, Haosen Ni, Yanbin Zheng, Ze Wang, Guang-Heng Dong

**Affiliations:** Department of Psychology, Yunnan Normal University, Kunming, 650500 Yunnan Province, China; Institute of Psychological Science, Hangzhou Normal University, Hangzhou, 310000 Zhejiang Province, China; Institute of Psychological Science, Hangzhou Normal University, Hangzhou, 310000 Zhejiang Province, China; Department of Psychology, Yunnan Normal University, Kunming, 650500 Yunnan Province, China; Department of Psychology, Yunnan Normal University, Kunming, 650500 Yunnan Province, China; Department of Psychology, Yunnan Normal University, Kunming, 650500 Yunnan Province, China; Institute of Psychological Science, Hangzhou Normal University, Hangzhou, 310000 Zhejiang Province, China; Department of Diagnostic Radiology and Nuclear Medicine, University of Maryland School of Medicine, Baltimore, MD 21201, USA; Department of Psychology, Yunnan Normal University, Kunming, 650500 Yunnan Province, China

**Keywords:** tobacco use disorder, repetitive transcranial magnetic stimulation, cerebral blood flow, functional connectivity, left dorsolateral prefrontal cortex

## Abstract

Repetitive transcranial magnetic stimulation (rTMS) is a non-invasive brain stimulation method that has been increasingly used to treat psychiatric disorders, including tobacco use disorder. However, the neural mechanisms underlying the effects of rTMS remain unclear. This study aimed to examine the effectiveness of rTMS in smoking cessation and to explore the underlying neural mechanism of the treatment effect. In Experiment 1, we recruited 60 participants who smoked cigarettes and 60 healthy controls and used their baseline cerebral blood flow (CBF) measured by arterial spin labelling perfusion to determine the group-level difference in CBF. In Experiment 2, we used the left dorsolateral prefrontal cortex (DLPFC) as the target for subsequent 5-day rTMS treatment at a frequency of 10 Hz with 2000 pulses to observe the impact of rTMS on CBF, Fagerström test for nicotine dependence scores and Tiffney questionnaire on smoking urges scores. In Experiment 3, we measured functional connectivity to monitor the functional changes induced by rTMS and assessed their associations with smoking cravings and nicotine dependence scores. In Experiment 1, participants who smoked cigarettes presented significantly higher CBF in the left DLPFC and bilateral anterior cingulate cortex than healthy controls. In Experiment 2, rTMS significantly decreased CBF in the DLPFC and reduced Fagerström test for nicotine dependence scores and Tiffney questionnaire on smoking urges scores. In Experiment 3, rTMS increased functional connectivity between the left DLPFC and the bilateral superior frontal gyrus, right DLPFC, bilateral precuneus and bilateral parahippocampus in participants, who smoked cigarettes. Regional CBF is a tool to identify tobacco use disorder-related regional brain markers and targets for reducing nicotine dependence and smoking cravings through rTMS. A neural mechanism of left DLPFC rTMS may involve a reduction in CBF in the target area and an increase in functional connectivity between the target area and the DLPFC–striatal pathways.

## Teaser

Li *et al*. explored the potential neural mechanisms of repetitive transcranial magnetic stimulation over the left dorsolateral prefrontal cortex for smoking cessation. They reported that repetitive transcranial magnetic stimulation may work by reducing cerebral blood flow in the target region and increasing functional connectivity with fronto-striatal pathways.

## Graphical Abstract

**Graphical Abstract:**
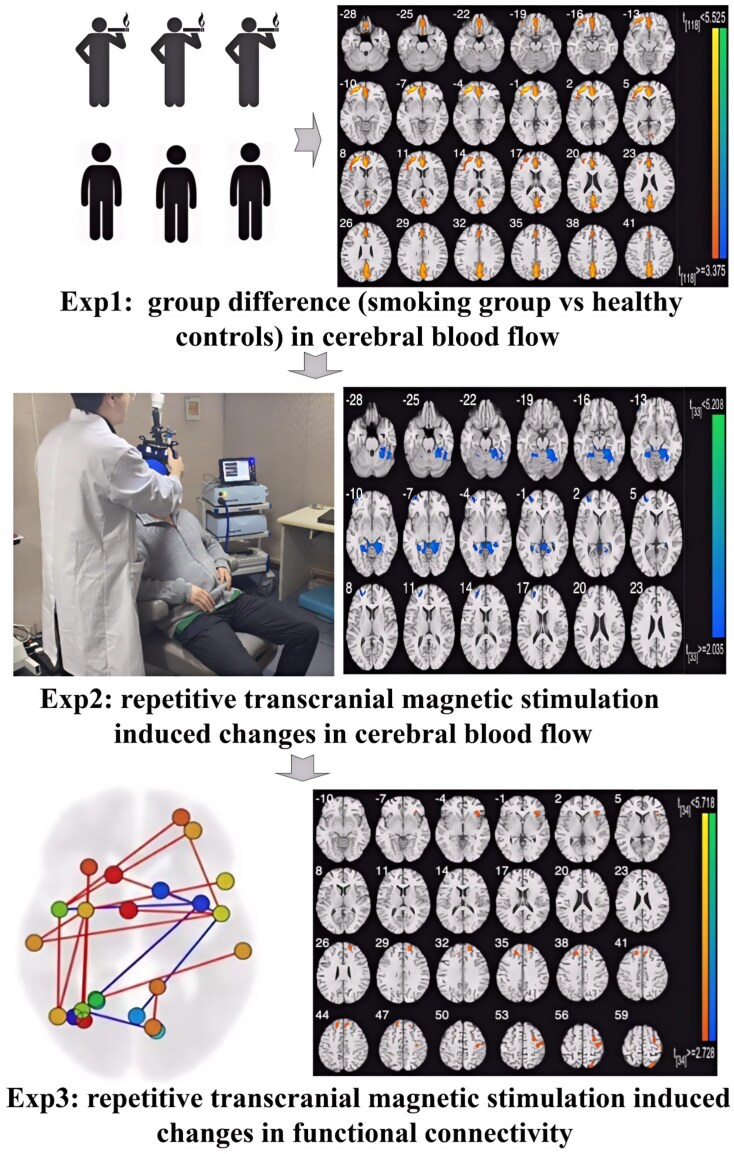

## Introduction

Tobacco use disorder (TUD) is a leading cause of preventable death globally.^1^ TUD is believed to cause the death of almost 6 million people annually^2^ because it increases the risk of a wide range of health conditions, including lung cancer, chronic bronchitis, emphysema and other serious diseases.^3^ Nicotine affects the vascular system, leading to a change in cerebral blood flow (CBF)^4,5^ and increased the risks of cognitive impairment and vascular dementia.^6^ TUD is characterized by cravings and withdrawal, compulsive use despite the negative consequences and repeated relapses,^7-10^ further exacerbating its public health burden. Understanding the mechanisms underlying TUD’s impact on brain are crucial for developing targeted and effective interventions.

Several smoking treatments or medicines have been developed in recent decades, including nicotine replacement therapy, varenicline and psychotherapy. However, all of them exhibit a low abstinence rate of ∼25%.^11,12^ While current treatments may alleviate withdrawal symptoms, their impact on the neural circuits underlying addiction remains limited. Therefore, a more comprehensive understanding of the neural mechanisms driving dysfunction in the brain networks associated with nicotine addiction is crucial. In this context, repetitive transcranial magnetic stimulation (rTMS) presents a promising alternative, with the potential to address some of the challenges associated with current smoking cessation therapies.

rTMS is a non-invasive neuromodulation approach that has been shown to be able to alter brain activity. TMS can be applied to a focal brain region.^13^ The US Food and Drug Administration (FDA) has cleared rTMS for short-term smoking cessation in adult smokers.^14^ Clinical studies have shown that high-frequency rTMS over the left dorsolateral prefrontal cortex (DLPFC) effectively decreases smoking cravings and cigarette intake.^15^ For example, according to the competing neurobehavioural decision system model, chronic nicotine use can disrupt the balance between the executive function network and impulsive network, leading to altered decision-making processes.^16,17^ By stimulating the left DLPFC with rTMS, reward decision-making is affected, which in turn reduces the craving for immediate rewards such as smoking.^18^ The majority of existing studies have focused on the impact of rTMS on cravings using behavioural measures.^19^ However, the neural mechanisms underlying the effects of rTMS are still unclear.

Due to its roles in executive functions, the inhibition of drug-related cravings and self-regulation, the DLPFC has been identified as a primary target in most studies.^20,21^ The mechanism by which high-frequency rTMS targeting the left DLPFC aids in smoking cessation may involve remote effects of rTMS. Remote effects mean that rTMS can not only directly influence functional connectivity (FC) within the targeted network but also modulate the FC of brain regions distal to the target area.^22,23^ Resting-state functional connectivity (rsFC) refers to the temporal correlations in neural activity between different regions of the brain when the individual is at rest.^24^ FC provides complementary insights to CBF by capturing the interaction between brain networks. For example, rTMS over the left DLPFC is likely to influence the resting-state brain activity and FC of the insula in people who smoke cigarettes.^25,26^ Additionally, studies have shown that rTMS over the left DLPFC can reduce local brain activity in the right insula and thalamus and decrease FC between the left DLPFC and the left orbitofrontal medial prefrontal cortex in people who smoke cigarettes.^26^ In this study, FC was included alongside CBF to provide a broader picture of the network-level changes induced by rTMS, aiming to capture both localized brain activity and brain network interactions.

Arterial spin labelling (ASL), a non-invasive magnetic resonance imaging (MRI) tool, can measure CBF using magnetically labelled arterial blood water as an endogenous tracer.^27,28^ CBF is coupled with regional brain function and has been used to probe alterations in regional brain function in people who chronically smoke cigarettes during cue-induced nicotine craving^5,29^ and withdrawal-induced cigarette craving.^5^ CBF could reveal brain activity linked to smoking craving. Several studies have shown that CBF in the posterior cingulate and dorsolateral prefrontal cortices,^30^ as well as in the anterior cingulate and hippocampus,^31^ is positively correlated with craving scores. Another study reported that increasing nicotine craving scores predict an increase in CBF in the frontal lobes, thalamus and striatum.^5^ These studies showed the potential of ASL CBF for identifying alterations in regional function associated with cigarette craving, but they were limited by the moderate sample size and the use of the suboptimal ASL imaging sequences available at that time. Our study differed in its use of a larger sample size and the state-of-art ASL MRI sequence recommended by the ASL research community.^32^

The goals of this study were 2-fold: we first aimed to explore the brain regions that exhibit differences in CBF between participants who smoked cigarettes and healthy controls; we then aimed to explore whether a 5-day rTMS treatment can reduce smoking cravings and to examine the potential neural mechanism of rTMS by assessing both the changes in CBF and FC.

## Materials and methods

### Ethics

All procedures contributing to this work comply with the ethical standards of the relevant national and institutional committees on human experimentation and with the 1975 Declaration of Helsinki and its revision in 2008. The Human Investigations Committee of Hangzhou Normal University approved this research with approval No. 20190505. The protocol trial was registered at the Chinese Clinical Trial Registry (ChiCTR2300076233 at: http://www.chictr.org.cn). The participants were recruited through advertisements. All participants provided written informed consent before the experiment/scan.

### Inclusion and exclusion criteria for participants

The participants who smoked cigarettes had to be motivated to quit (replying ‘very likely’ or ‘somewhat likely’ to a motivation questionnaire) and had no period of abstinence of more than 3 months in the past year. The criteria for participants who smoked cigarettes were as follows: (i) smoked at least ten cigarettes per day for 1 year or more; (ii) had carbon monoxide (CO) levels in expired air of at least 5 ppm measured using the Smokerlyzer System; (iii) had a cigarette dependence score higher than four, as evaluated by the Fagerström test for nicotine dependence (FTND)^33,34^; and (iv) had a score of more than 15 on the 10-item Tiffney questionnaire on smoking urges (TQSU).^35^

The criteria for healthy control (HCs) were as follows: (i) participants must have never smoked or must have been smoke-free for at least 5 years prior to the study; (ii) participants should not have a history of nicotine use or dependence; (iii) participants should not use any nicotine-containing products, such as e-cigarettes, nicotine gums, or patches; and (iv) participants must be free from any psychiatric disorders, particularly those related to substance abuse.

All participants (who smoked cigarettes and healthy controls) provided satisfactory answers to a safety screening questionnaire for rTMS and functional magnetic resonance imaging (fMRI), which included the absence of all of the following: (i) any mental or neurological-related disease or related history; (ii) cognitive impairment according to the Mini-International Neuropsychiatric Interview (MINI)^36^ or depression according to the Beck Depression Scale^37,38^; (iii) surgery, head trauma or heart-related diseases in the past year; (iv) claustrophobia; (v) metal implants or tattoos of the neck or head; (vi) any other substance use disorder during the last 12 months before recruitment; (vii) use of any psychotropic medication on a regular basis; and (viii) a history of epilepsy or seizures or increased risk of seizures for any reason.

**Figure 1:**
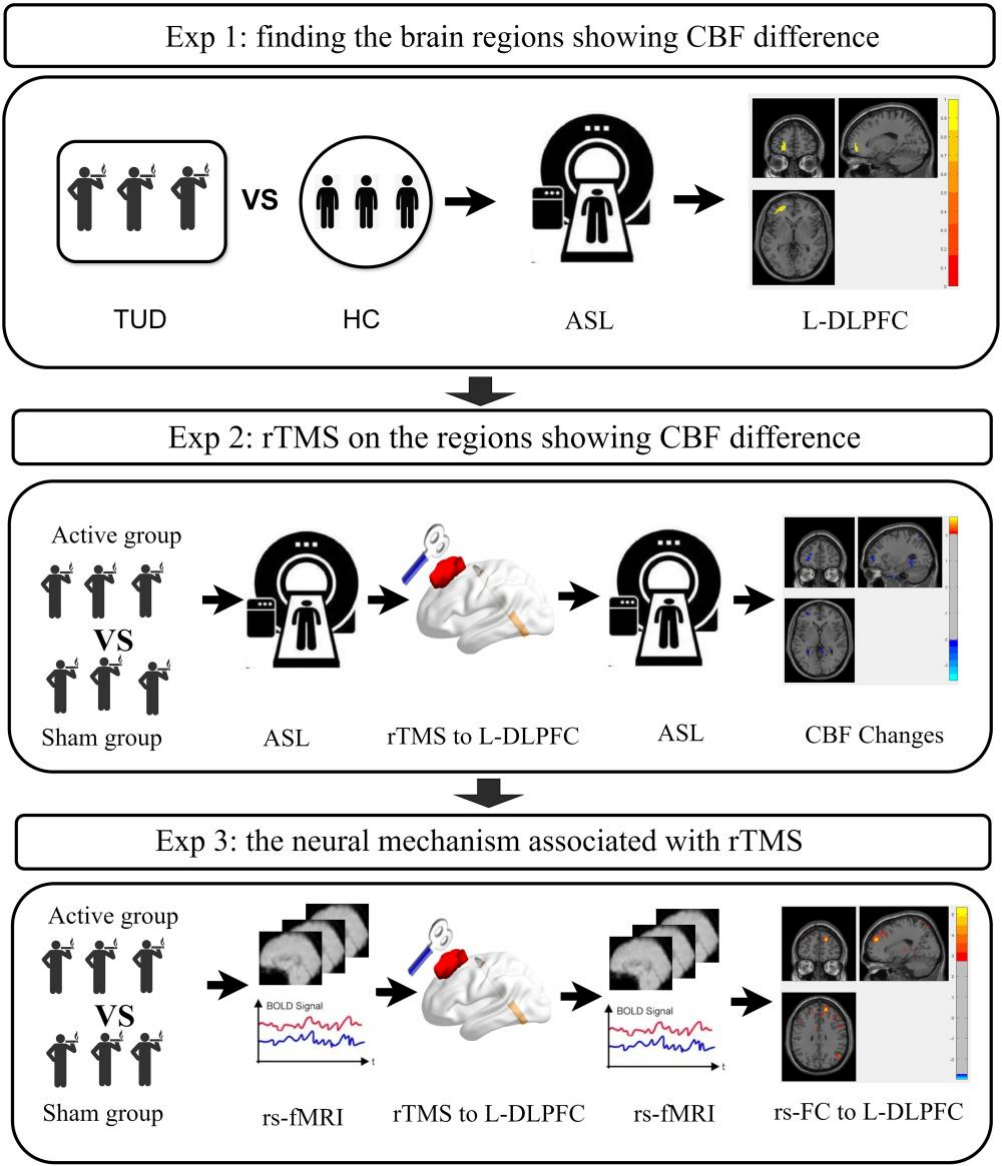
**The experimental procedure used in the three consecutive studies.** In Experiment 1, we explored the brain regions showing differences in CBF between participants who smoked cigarettes and healthy controls. In Experiment 2, we performed rTMS of the brain regions showing group differences [left dorsolateral prefrontal cortex (L-DLPFC)]. In Experiment 3, we further investigated the neural mechanism associated with rTMS treatment. CBF, cerebral blood flow; TUD, tobacco use disorder; HC, healthy control; ASL, arterial spin labelling; L-DLPFC, left dorsolateral prefrontal cortex; rTMS, repetitive transcranial magnetic stimulation; rs-fMRI, resting-state functional magnetic resonance imaging; rsFC, resting-state functional connectivity.

Figure 1 provides an overview of the entire study. The top panel shows the cross-sectional design for the baseline CBF comparison study for identifying the difference in regional CBF between the participants who smoked cigarettes and HCs; the middle panel shows the rTMS treatment.

### Experiment 1

#### Participants

Sixty healthy male controls and sixty male participants who smoked cigarettes seeking treatment participated in the study. The participants who smoked cigarettes were instructed to maintain their usual smoking habits and abstain from smoking for 1 h before the fMRI scan. The 1-h abstinence period was due to the necessary safety checks and preparation that participants had to complete after entering the lab and before the scan began. Similarly, healthy controls also underwent the same safety checks and preparation, resulting in an equivalent 1-h waiting period before the scan. Detailed demographic information is listed in Table 1.

**Table 1:** The demographic information for participants in Experiment 1

	Participants who smoked cigarettes		Healthy controls	
	M	SD	M	SD
Age	23.31	4.72	24.72	4.09
Smoking age	5.72	4.55		

#### Data acquisition

All participants underwent resting-state scans for ASL data acquisition. MRI images were acquired using a GE Healthcare MR-750 3-T scanner with an eight-channel head coil at the Centre for Cognition and Brain Disorders of Hangzhou Normal University. Straps and foam pads were used to minimize head movement during the scan.

ASL MRI data were acquired using the GE product ASL sequence, which is a 3D background-suppressed stack of spiral readout-based pseudocontinuous ASL sequences. The imaging parameters were as follows: repetition time (TR)/echo time (TE) = 4816/57.5 ms, spiral readout = 12 arms × 512 samples, field of view = 22 cm, slice thickness = 3.0 mm, number of excitations (NEX) = 3, labelling duration = 1500 ms and post-labelling delay = 1525 ms. The duration of the resting-state scan was 6 min 33 s.

#### Data processing

Data were pre-processed using RESTPLUS, including realignment, smoothing, coregistration, default masking and normalization of perfusion parametric maps to the standard space of the Montreal Neurological Institute (MNI) brain. Spatial smoothing was conducted using an isotropic Gaussian filter with a full width at half maximum (FWHM) of 6 mm^3^. CBF maps were calculated via an statistical parametric mapping (SPM)-based ASL data processing toolbox.^39^ The CBF maps were created by subtracting the labelled images from the control images via simple subtraction. The CBF value was calculated using the following equation^32,40^:


$$\mathrm{CBF}(\mathrm{ml}/100\;g/\min)=\frac{6000\lambda \Delta MR}{2\alpha M_{0}\{\exp(-wR)-\exp[-(\tau +w)R]\}},$$

where Δ*M* is the ASL perfusion difference, *λ* (0.9 g/ml) is the blood–tissue water partition coefficient, *R* (0.67 s^−1^) is the longitudinal relaxation rate of blood, *α* is the tagging efficiency, *M*_0_ is the equilibrium magnetization of the brain, *w* is the post-labelling delay and *τ* is the duration of the labelling radio frequency pulse train.

#### Statistical analyses

Differences in brain activity between participants who smoked cigarettes and healthy controls were determined using two-sample *t*-tests (two-tailed) of the mean CBF data. The significance thresholds for the two-tailed *t*-tests were set at *P* < 0.005 based on AlphaSim to correct for multiple comparisons over the whole brain.

#### Results

**Figure 2:**
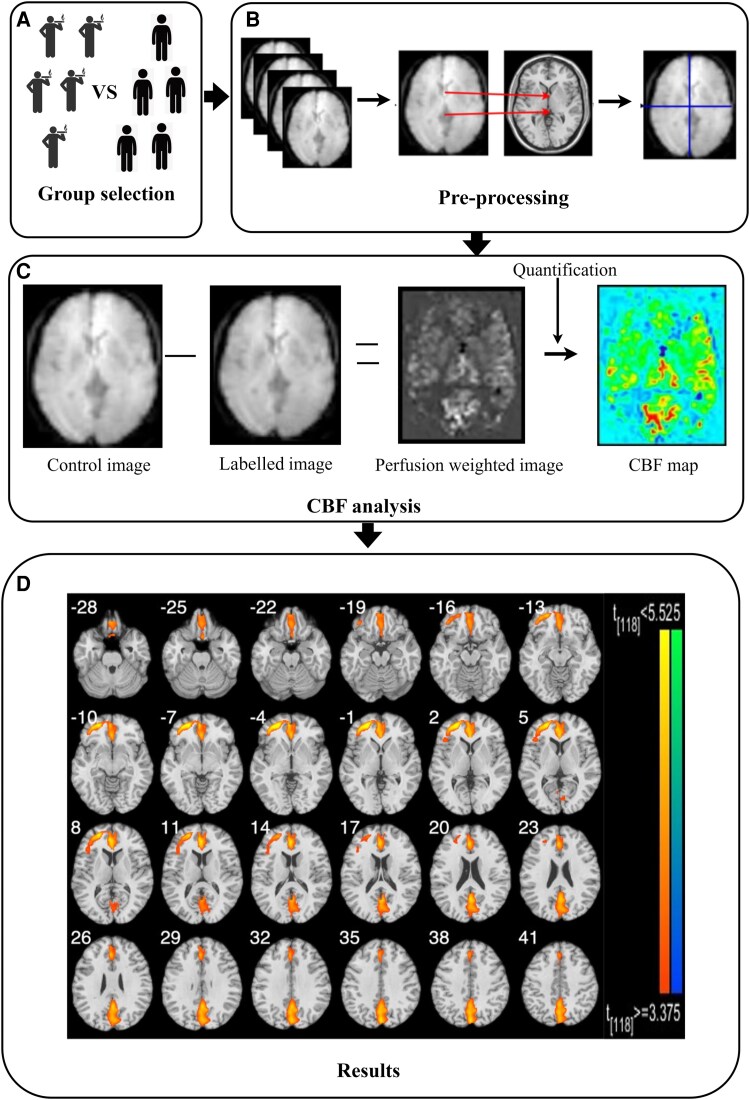
**The data analysis procedure and results of Experiment 1.** (**A**) Sixty healthy controls and sixty participants who smoked cigarettes participated in the study. (**B**) Data pre-processing procedure, including motion correction, coregistration and smoothing. (**C**) Calculation of the CBF value. (**D**) Using two-sample *t*-tests (two-tailed), we observed that the left DLPFC, bilateral anterior cingulate and bilateral precuneus had greater CBF in participants who smoked cigarettes than in healthy controls (*P* < 0.005). CBF, cerebral blood flow; DLPFC, dorsolateral prefrontal cortex.

The left DLPFC (LH-DLPFC; cluster size *k* = 511, MNI coordinates *x* = −29, *y* = 48, *z* = −1), bilateral anterior cingulate (LH-ACC cluster size *k* = 875, MNI coordinates *x* = −1, *y* = 39, *z* = 12; RH-ACC cluster size *k* = 586, MNI coordinates *x* = 4, *y* = −66, *z* = 19) and bilateral precuneus (LH-precuneus cluster size *k* = 487, MNI coordinates *x* = 1, *y* = −67, *z* = 43; RH-precuneus cluster size *k* = 523, MNI coordinates *x* = 2, *y* = −57, *z* = 48) had greater CBF in participants who smoked cigarettes than in healthy controls (Fig. 2).

#### Discussion of Experiment 1

We utilized ASL perfusion fMRI to evaluate differences in resting-state brain activation between participants who smoked cigarettes and healthy controls. CBF can serve as an indicator of brain activation.^41^ In our study, we observed a significant increase in CBF in the left DLPFC, bilateral ACC and bilateral precuneus of participants who smoked cigarettes compared with healthy controls, suggesting that nicotine affects neural activity in these regions. Partially consistent with our findings, previous studies have reported an increase in CBF in the ACC/medial orbitofrontal cortex (OFC) and the left OFC has been reported in people who smoke cigarettes.^5^ In our study, we specifically observed an increase in CBF in the ACC. One possible explanation is that the ACC is a brain region associated with reward, and increased CBF in this area may be linked to increased processing of reward signals in the brain.^5,42-44^ However, we did not observe significant changes in the medial or left OFC. This discrepancy could be attributed to differences in study design, participant characteristics, or methodological approaches. Further studies are warranted to explore the potential variability in CBF changes across these brain regions in relation to smoking. The DLPFC is involved in behavioural control, and the increase in CBF in this region may indicate a heightened motivation to regulate smoking behaviours.^5^ Additionally, another study demonstrated that people who smoke cigarettes showed significantly greater activation in the precuneus and supramarginal gyrus.^45^ The precuneus is a key part of the default mode network, a network that is active during rest and is involved in functions such as self-awareness and reward-related memories.^46^ Increased neural activity in the precuneus may be associated with heightened smoking-related memories and cravings.^47^ This increase in CBF across the DLPFC, ACC and precuneus reflects a complex interplay of cognitive control and reward processing mechanisms in people who smoke cigarettes, suggesting the brain's efforts to manage cravings.

### Experiment 2

In Experiment 1, we found that the brain region showing differences in participants who smoked cigarettes was the left DLPFC. Based on these findings, we performed a 5-day rTMS treatment of the left DLPFC to observe the changing pattern of CBF before and after rTMS and evaluate the effectiveness of rTMS in smoking cessation.

#### Participants

The participants were the 60 males who smoked cigarettes from Experiment 1. During the rTMS intervention, 7 participants dropped out before completing the full 5-day treatment, resulting in a final sample of 53 participants who smoked cigarettes who completed the entire study (overall retention: 88%). We randomly divided the participants into two groups. Finally, 35 participants in the active group and 18 participants in the sham group completed the entire treatment procedure and were included in the final analysis. The participants were required to maintain their usual smoking habits and refrain from smoking for 1 h before the fMRI scan. Detailed demographic information can be found in Table 2.

**Table 2:** Demographic information for the participants in Experiments 2 and 3

	Active group				sham group			
	Pre		Post		Pre		Post	
	M	SD	M	SD	M	SD	M	SD
Age	23.03	3.54			22.41	2.37		
Smoking age	5.32	3.30			4.27	1.67		
Fagerström test for nicotine dependence	4.63	1.77	3.29	1.84	4.39	1.91	3.94	1.83
Tiffney questionnaire on smoking urges	25.43	9.28	20.29	9.98	27.22	8.93	26.17	8.73

#### Design

We employed a 2 (group: active versus sham) × 2 (time: pre-rTMS treatment versus post-rTMS treatment) mixed experimental design with a single-blind procedure. In the single-blind phase, participants were randomly assigned to either the active rTMS group or the sham rTMS group using a randomized sequence method. In the sham group, the TMS coil was positioned vertically at the target site and the machine was activated to replicate the sensory experience (e.g. sound and tactile sensations) of the active rTMS treatment, ensuring that participants felt they were being stimulated. However, no therapeutic stimulation was delivered to the brain, as the coil was placed at an angle to avoid the magnetic field’s effects on brain activity. All other procedures, including the timing, frequency and duration of the treatment, were identical to those in the active group to maintain consistency across both groups. Participants remained blinded throughout the study, with no information provided about their group allocation, ensuring that their expectations did not influence the results.

**Figure 3:**
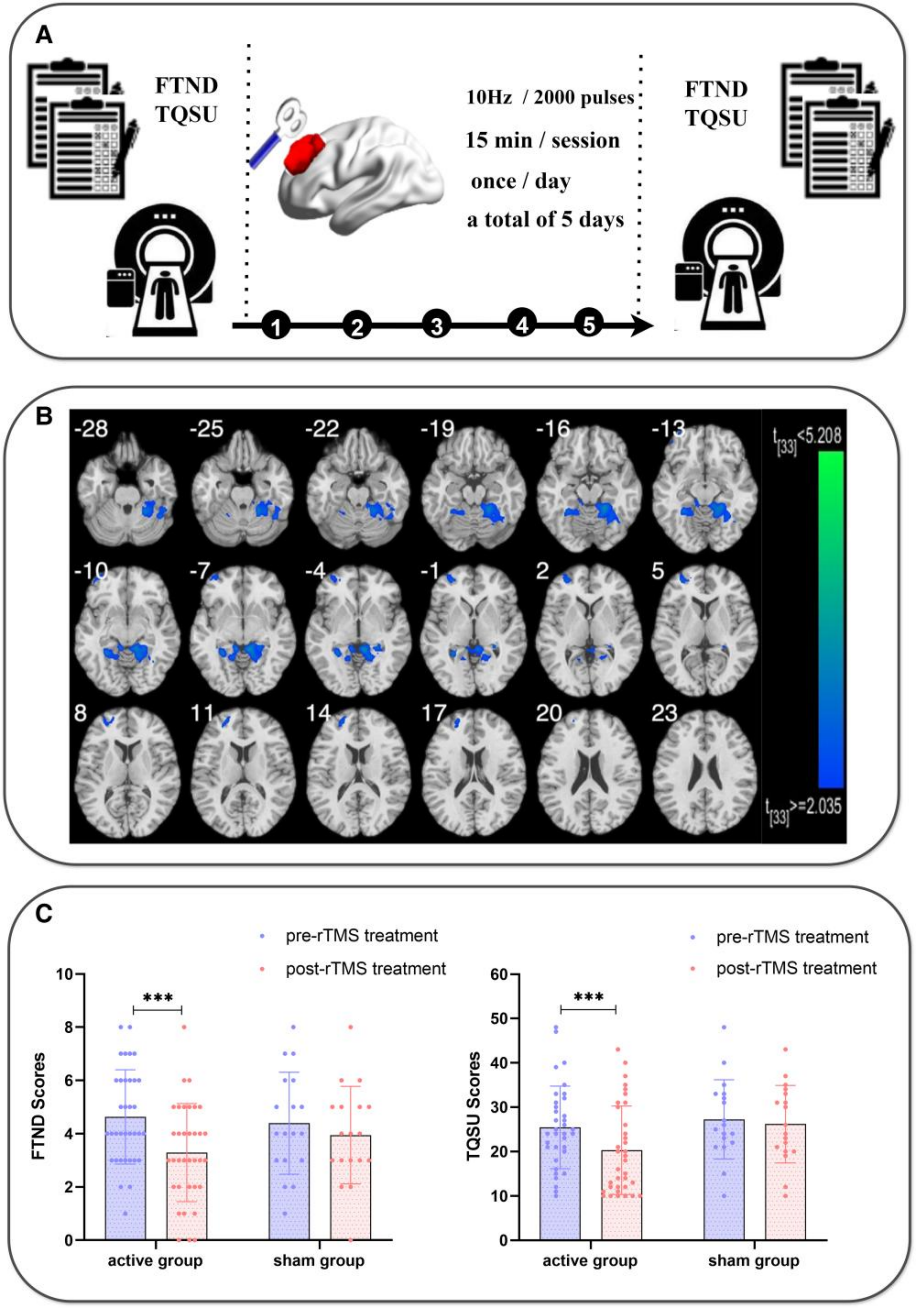
**Experimental procedure and results of Experiment 2.** (**A**) The participants who smoked cigarettes underwent fMRI scans and completed the FTND and TQSU scales. Then, they underwent 5 days of rTMS treatment. A stimulation session consisted of 40 trains of 10 Hz stimulation, each lasting for 5 s with an interval of 10 s. Each treatment session lasted for 15 min, with a total of 2000 pulses. Finally, the participants underwent another round of fMRI scans and completed the FTND and TQSU scales. (**B**) Panel B represents brain activation maps derived from CBF data. In this study, each data point corresponds to a voxel within the brain region analysed. The experimental unit is the individual participant, as the imaging data were collected on a per-participant basis. For the specific analysis shown in **B**, the maps were generated based on a sample size of (*n* = 53) participants. Using paired *t*-tests (two-tailed), we found that the CBF in the left DLPFC and bilateral hippocampus was lower after rTMS treatment than before rTMS treatment (*P* < 0.005). (**C**) The experimental unit is also the individual participant. Each data point represents the FTND and TQSU score of a single participant before (pre-rTMS) and after (post-rTMS) treatment. The sample size for the active group and sham group is (35, 18). Paired *t-*tests confirmed that the post-treatment FTND and TQSU scores (FTND: 3.29 ± 1.84; TQSU: 20.29 ± 9.98) were significantly lower than the pre-treatment scores (FTND: 4.63 ± 1.77; *t* = 5.71, *P* < 0.001; TQSU: 25.43 ± 9.28; *t* = 4.74, *P* < 0.001) in the active group. ***Indicates a *P*-value less than 0.001. FTND, Fagerström test for nicotine dependence; TQSU, Tiffney questionnaire on smoking urges; rTMS, repetitive transcranial magnetic stimulation; fMRI, functional magnetic resonance imaging; CBF, cerebral blood flow; DLPFC, dorsolateral prefrontal cortex.

The timeline for treatment and assessments is provided in Fig. 3. Participants’ baseline measurements were taken on the first day, including the FTND score, TQSU score and fMRI scans, as well as the safety assessments for rTMS. During fMRI, participants underwent pseudocontinuous arterial spin labelling to measure brain perfusion. From the second to the sixth days, rTMS treatment was administered for five consecutive days. The FTND score, TQSU score and fMRI scans were also measured on the seventh day.

An rTMS research system with a 70-mm biphasic figure-8 coil and a special air-cooling system was used in a room close to the MRI scanner room.

We determined the resting motor threshold (RMT) of each participant at the beginning of each experimental visit prior to any exposure or ratings. Individual RMTs were determined by positioning the coil 45° above the area of the skull corresponding to the contralateral motor cortex. We then adjusted the amplitude of the magnetic pulse during each single pulse until we determined the lowest intensity that reliably produced thumb or hand movements in at least 5 of 10 consecutive trials. We placed the Cz point of the 10–20 electroencephalography system at the central location, then we leaned ∼60° the left and the M1 area was determined ∼1 cm ahead. The left DLPFC was located by moving the coil 5 cm anterior to M1 and we marked the location using a special hat to maintain the same location of stimulation in each participant.^48,49^

Treatment was standardized at 100% RMT. Each participant underwent a continuous 5-day treatment. A stimulation session consisted of 40 trains of 10 Hz with 2000 pulses, each lasting for 5 s with an interval of 10 s. Each treatment session lasted for 15 min, which included ∼10 min of direct stimulation time and an additional 5 min for setup and equipment adjustments.

#### Data processing

Data pre-processing, including realignment, smoothing, coregistration, default masking and normalization of perfusion parametric maps to the standard space of the MNI brain, was performed using RESTPLUS. Spatial smoothing was conducted using an isotropic Gaussian filter with a FWHM of 6 mm^3^. CBF maps were calculated via an SPM-based ASL data processing toolbox.^39^ The CBF maps were created by subtracting the labelled images from the control images via simple subtraction.

#### Statistical analyses

Changes in brain activity in participants who smoked cigarettes before and after treatment were determined by two-tailed paired *t*-tests, in which significance thresholds were set at *P* < 0.005 based on AlphaSim to correct for multiple comparisons over the entire brain.

#### Results

##### CBF results

The CBF in the left DLPFC (LH-DLPFC; cluster size *k* = 97, MNI coordinates *x* = −24, *y* = 67, *z* = 54) was lower after rTMS than before rTMS (Fig. 3). Compared with rTMS, a decrease in the CBF of the bilateral hippocampus (LH-hippocampus, cluster size *k* = 129, MNI coordinates *x* = −27, *y* = −44, *z* = −4; RH-hippocampus, cluster size *k* = 135, MNI coordinates *x* = 27, *y* = −39, *z* = −3) associated with reward circuits was also observed. Additionally, CBF in the bilateral culmen (LH-culmen, cluster size *k* = 55, MNI coordinates *x* = −9, *y* = −39, *z* = −6; RH-culmen, cluster size *k* = 124, MNI coordinates *x* = 21, *y* = −42, *z* = −15) and the right occipital lobe (RH-occipital lobe, cluster size *k* = 82, MNI coordinates *x* = 30, *y* = −54, *z* = −15) were lower after rTMS than before rTMS. No significant difference was observed in the sham group (Fig. 3).

##### Behavioural results

Paired *t*-tests confirmed that the post-treatment FTND score (3.29 ± 1.84) was significantly lower than the pre-treatment score (4.63 ± 1.77; *t* = 5.71, *P* < 0.001) in the active group. Furthermore, a significant reduction in the post-treatment TQSU score (20.29 ± 9.98) was observed compared with the pre-treatment score (25.43 ± 9.28; *t* = 4.74, *P* < 0.001) in the active group. No difference was observed in the sham group (Fig. 3).

#### Discussion of Experiment 2

After a 5-day treatment period, we observed a reduction in CBF in both the left DLPFC and hippocampus of participants who smoked cigarettes, in addition to concurrent decreases in the FTND and TQSU scores. A previous study revealed that significant decreases in nicotine cravings and CBF were observed after 10 days of rTMS treatment,^50^ which was consistent with our study. The reduction in CBF in the DLPFC may indicate that rTMS treatment reduces overactivation in this brain region.^50^ Specifically, in Experiment 1, we found that, compared with healthy controls, participants who smoked cigarettes presented greater CBF in the DLPFC. This elevated activity in the DLPFC may reflect an attempt to exert additional regulatory control within an imbalanced neural network. Such compensatory activation could indicate that the DLPFC is working harder to maintain cognitive or behavioural regulation in the context of disrupted neural functioning associated with smoking. However, this heightened demand on the DLPFC may also signal an inefficient allocation of neural resources, contributing to the difficulty in regulating smoking behaviour. By modulating activity in these regions, rTMS may help restore balance within the neural network, thereby reducing the excessive demand on the DLPFC. This is reflected in the observed decrease in CBF, which could indicate a normalization of brain function. Such changes highlight the therapeutic potential of rTMS in alleviating the neural dysregulation underlying smoking behaviour and supporting smoking cessation. Several studies have shown that rTMS of the DLPFC can effectively reduce smoking cravings,^19,51-53^ which was confirmed by the decreases in the FTND and TQSU scores reported in our study. The hippocampus is a brain region associated with nicotine-related reward memories. The formation of these reward memories, which link smoking behaviour to feelings of pleasure, is a crucial psychological foundation for nicotine cravings.^31^ Studies have shown that nicotine enhances the stability of these smoking-related memories by affecting synaptic plasticity in the hippocampus,^54^ thereby intensifying cravings and increasing the risk of relapse.^55,56^ Among smokers, changes in CBF in the hippocampus have been closely linked to nicotine cravings.^31^ In our study, rTMS may have modulated the neural mechanisms associated with these cravings by altering hippocampal activity. Specifically, rTMS might reduce neural activity in the hippocampus, thereby weakening the reward memories associated with smoking and decreasing cravings, as reflected in the observed reduction in CBF. These results provide evidence to support the effectiveness of rTMS of the left DLPFC in smoking cessation.

### Experiment 3

Compared with that of CBF, the blood oxygen level dependent (BOLD) signal of fMRI is more reliable in spatial network patterns^57^; thus, we also acquired BOLD resting-state functional MRI data before and after rTMS treatment. Additionally, both the BOLD and CBF data were acquired during the same imaging session to ensure consistency and reduce variability in the data collection process. The rTMS treatment procedure was the same as that in Experiment 2 (Fig. 3). We employed a seed-based rsFC approach in Experiment 3 to further investigate the neural connectivity between the left DLPFC and other key networks involved in addiction, especially given the observed deactivation between the DLPFC and hippocampus in Experiment 2.

#### Participants

The same group of participants included in Experiment 2 were analysed.

#### Imaging data acquisition and data processing

Magnetic resonance images were acquired on a 3.0-T whole-body GE 750 MR scanner (GE, Milwaukee, USA) using a standard 8-channel receiver array. The structural images were acquired using a T_1_-weighted inversion with an field of view = 25.6, TR/TE = 7/3, slice thickness = 1.0, flip angle = 7 and bandwidth = 31.25.

All imaging data pre-processing and analyses were performed using statistical parametric mapping (SPM12) within the MATLAB R2017b environment. Data pre-processing was performed using the RESTPLUS toolboxes and the DPABI toolboxes were utilized for the statistical analysis of the data. Specifically, each subject’s images were slice–time corrected, realigned and coregistered with the anatomical image of each subject. White matter, CSF and global mean signals were regressed out of the images. Finally, the images were spatially normalized to the MNI space and smoothed with a Gaussian Kernel with an FWHM of 6 mm. Smoothed normalized images were used for seed-based region of interest (ROI) analyses.

#### Statistical analysis

A seed-based correlation analysis was employed to examine the FC between the left DLPFC and other brain regions. A 6-mm diameter sphere centred at the MNI coordinates of (−30, 54, 3) was defined as a seed region.^58,59^ FC analyses were performed at the voxel-wise level, and differences before and after rTMS treatment were detected in the sham group and active group using paired *t-*tests (two-tailed). Significance thresholds for the two-tailed *t-*tests were set at corrected *P* < 0.01 based on AlphaSim to correct for multiple comparisons over the entire brain. The cluster volumes are reported in cubic millimetres.

#### Results

Compared with pre-treatment measurements of participants who smoked cigarettes, post-treatment measurements of participants who smoked cigarettes revealed increased FC between the left DLPFC and the bilateral superior frontal gyrus (SFG), right DLPFC, bilateral precuneus and bilateral parahippocampus (Table 3). No significant differences were detected in the sham group.

**Table 3:** Brain areas with significant differences in the functional connectivity of the left DLPFC between post-treatment and pre-treatment

Brain region	Brodmann area	Volume (mm^3^)	Coordinates (*x*, *y*, *z*)
Right superior frontal gyrus	8/10	81	23, 49, 27
Left superior frontal gyrus	8	89	−19, 38, 39
Right dorsolateral prefrontal cortex	6/8/9	139	33, −3, 53
Right precuneus	7	69	24, −72, 50
Left precuneus	4	67	−10, −35, 58
Left parahippocampal	Hippocampus	45	−27, −32, −4
Right parahippocampal	30	41	24, −39, 1

**Figure 4:**
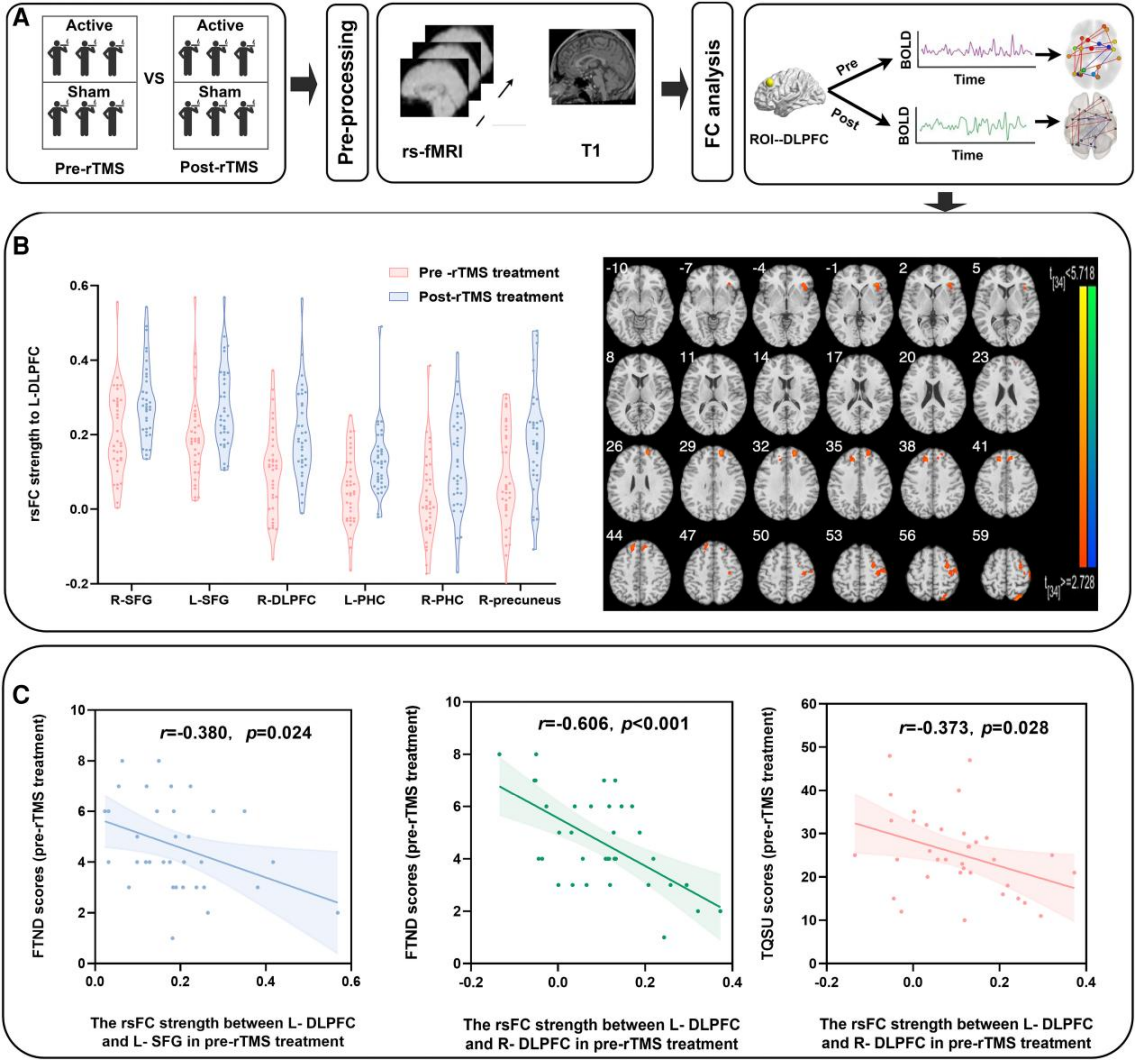
**Data analysis procedure and results of Experiment 3.** (**A**) The data analysis methodology comprises pre-processing and statistical analysis. (**B**) The experimental unit is also the individual participant. The data points represent Fisher’s *Z*-transformed correlation coefficients, which quantify the FC strength between the L-DLPFC and specific brain regions (e.g. R-SFG and L-SFG). The data points in the figure display the pre-treatment and post-treatment rsFC values, respectively. The sample size for this analysis is *n* = 53, with all participants contributing both pre- and post-treatment rsFC values. Paired *t*-tests (two-tailed) revealed that, compared with pre-treatment assessments of participants who smoked cigarettes, post-treatment assessments of participants who smoked cigarettes presented increased FC between the left DLPFC and bilateral superior frontal gyri, right DLPFC, bilateral precuneus and bilateral parahippocampus (*P* < 0.01). (**C**) The experimental unit is the individual participant. Each data point represents the correlation value between the FC strength of a specific brain region and the FTND or TQSU scores for that participant. The sample size for this analysis is *n* = 53. Pearson’s correlation analysis revealed that the rsFC strength between the left DLPFC and the left SFG was significantly negatively correlated with the FTND scores before rTMS treatment (*r* = −0.380, *P* = 0.024); the rsFC strength between the left DLPFC and the right DLPFC was negatively associated with the FTND and TQSU scores before rTMS treatment (FTND: *r* = −0.606, *P* < 0.001; TQSU: *r* = −0.373, *P* = 0.028). rTMS, repetitive transcranial magnetic stimulation; rs-fMRI, resting-state functional magnetic resonance imaging; T_1_, T_1_-weighted imaging; rsFC, resting-state functional connectivity; ROI, region of interest; DLPFC, dorsolateral prefrontal cortex; BOLD, blood oxygen level dependent; R-SFG, right superior frontal gyrus; L-SFG, left superior frontal gyrus; R-DLPFC, right dorsolateral prefrontal cortex; L-PHC, left parahippocampal cortex; R-PHC, right parahippocampal cortex; FTND, Fagerström test for nicotine dependence; TQSU, Tiffney questionnaire on smoking urges.

Based on Pearson’s correlation coefficient, the rsFC strength between the left DLPFC and the left SFG was significantly negatively correlated with the FTND score before rTMS (*r* = −0.380, *P* = 0.024). Moreover, the rsFC strength between the left DLPFC and the right DLPFC was negatively associated with the FTND and TQSU scores before rTMS (*r* = −0.606, *P* < 0.001; *r* = −0.373, *P* = 0.028) (Fig. 4).

#### Discussion of Experiment 3

We used a seed-based rsFC approach to explore the neural connectivity between the left DLPFC and other brain regions associated with rTMS treatments.

First, we observed that after rTMS, rsFC was enhanced between the left DLPFC and right DLPFC and between the left DLPFC and bilateral SFG, which have been identified as key regions involved in the executive control network (ECN).^60-62^ The ECN plays a crucial role in executive functions such as decision-making and inhibitory control, both of which are often impaired in individuals with nicotine dependence.^60-62^ Importantly, our findings revealed that rTMS over the left DLPFC has the potential to restore and enhance FC within the ECN in individuals who smoke cigarettes. This increase in ECN connectivity observed after rTMS is consistent with previous research showing that rTMS can modulate brain networks involved in cognitive control, particularly the ECN.^63^ The improved connectivity within the ECN suggests that rTMS may bolster cognitive control processes, which are critical for regulating smoking behaviour.^19^ By strengthening the neural pathways within the ECN, rTMS could enhance these executive functions, leading to a reduction in smoking behaviour, as supported by studies showing the effectiveness of rTMS in reducing cravings and smoking behaviour.^51^ These findings suggest that rTMS may be an effective intervention for addressing the neural deficits underlying smoking addiction.

Second, we also observed increased rsFC between the left DLPFC and hippocampus. Previous studies have shown that individuals who smoke cigarettes exhibit diminished rsFC within circuits involving the limbic, insular and prefrontal cortical regions, which are crucial for reward processing. Notably, reduced connectivity between the insula and the ECN has been observed in people who smoke cigarettes compared with those who do not, with the hippocampus frequently implicated in this disrupted circuitry.^64-66^ Our results demonstrated that rTMS treatment targeting the left DLPFC could increase the strength of connectivity between the ECN and reward circuit, particularly increasing connectivity between the DLPFC and the hippocampus. This enhancement may bolster the ability of the brain to inhibit smoking cravings by rebalancing the neural pathways involved in reward processing and cognitive control. Furthermore, accumulating evidence suggests that the inclination towards drug-seeking behaviour may stem from a dysregulation of the intricate interplay between the dopaminergic reward system and the cortical circuit that governs inhibition and cognitive decision-making processes.^67^ Consistent with this result, our findings support the hypothesis that rTMS of the left DLPFC could reshape frontostriatal pathways, thereby enhancing the regulatory influence of the ECN over the reward system. This improved regulation may lead to a reduction in nicotine-seeking behaviour by restoring the balance between cognitive control and reward-driven impulses. These results underscore the therapeutic potential of rTMS in modulating the neural circuits underlying nicotine addiction and highlight its role in reducing smoking behaviour through targeted neuromodulation.

In addition, we observed increased rsFC between the left DLPFC and precuneus. The precuneus is a central hub within the brain and is known for its extensive connections to both cortical and subcortical regions, making it integral to various cognitive functions, including those associated with addiction. Studies have shown that increased activation levels in the precuneus are correlated with addiction-related symptoms, including substance misuse difficulties.^68,69^ Moreover, a study showed that rTMS treatment decreased cravings and increased FC between the left DLPFC and inferior parietal lobule,^70^ suggesting that the precuneus acts as the relay between the ECN and reward circuits, which is consistent with our findings. In the present study, we observed that increased FC between the DLPFC and precuneus was correlated with reduced cravings. This finding suggests that DLPFC–precuneus connectivity may reflect the enhanced control of the ECN over subcortical regions involved in reward processing, particularly the hippocampus and related structures. The precuneus, acting as an intermediary, may facilitate the transmission of regulatory signals from the prefrontal cortex (e.g. DLPFC) to these subcortical regions, thereby exerting an inhibitory effect on reward-related cravings.^71^ The observed increase in FC between the DLPFC and the precuneus may indicate an improved capacity for executive control over smoking cravings, which in turn could lead to a reduction in smoking behaviour. This result aligns with the notion that the precuneus not only integrates information from various cortical and subcortical areas but also plays a critical role in modulating the interaction between cognitive control networks and reward systems.^72^ By strengthening the neural pathways between the DLPFC and the precuneus, rTMS may enhance the ability of the brain to suppress cravings and regulate behaviour, providing a potential mechanism for its therapeutic effects on smoking cessation.

## General discussion

By performing three experiments, we explored the potential neurological mechanisms underlying the effects of high-frequency rTMS on smoking cessation. Specifically, our findings suggest that rTMS targeting the left DLPFC appears to reduce craving scores and normalize the elevated perfusion state observed in participants who smoke cigarettes. Additionally, rTMS treatment increased the FC within key neural circuits associated with craving regulation and cognitive control, which are often disrupted in individuals with TUD.^73,74^ These findings suggest its promise as a supplementary therapy to behavioural interventions for smoking cessation, particularly in modulating the neural mechanisms that underlie addiction.

The present study has several limitations. The first limitation was that females who smoke cigarettes were not investigated in the study. In China, the prevalence of females who smoke cigarettes is <1%, and most of them prefer to conceal their smoking status because of social stigmas.^75^ Thus, further studies could examine the effect of rTMS on females who smoke cigarettes. The second limitation was the relatively small sample size in Experiments 2 and 3, which was mainly attributed to the long treatment cycle, resulting in subject loss. Third, due to the necessary safety checks and preparation before the MRI scan, a 1-h gap existed between the participants’ last cigarette and the scan. The potential impact of this brief abstinence on brain activity remains unknown. Future studies could explore this process in greater depth.

## Conclusions

The current study revealed that rTMS of the left DLPFC decreased smoking cravings and CBF features in the DLPFC of participants who smoked cigarettes. In addition, rTMS of the left DLPFC was associated with increased FC within the ECN and reshaped the DLPFC–striatal pathways. In summary, rTMS of the left DLPFC has the potential to serve as an efficacious tool for smoking cessation. In addition, CBF could serve as a valuable means of quantifying treatment effects.

## Data Availability

The data stored in our lab-based network attachment system can be accessed at: http://QuickConnect.cn/others, ID: guests, PIN dong@123.COM. All people who are interested in it can download directly.
